# Knowledge user perspectives on integrated knowledge translation (iKT) in health interventions research for people with multiple sclerosis: a qualitative descriptive study

**DOI:** 10.1186/s40900-025-00763-7

**Published:** 2025-07-28

**Authors:** Gregory Feng, Robert Simpson, Nanette Lai, Mark Bayley, Dorothy Luong, Sarah Munce

**Affiliations:** 1 KITE Research Institute (Toronto Rehabilitation Institute), University Health Network, Toronto, Canada; 2 Department of Population Medicine, University of Guelph, Guelph, Canada; 3 Holland Bloorview Kids Rehabilitation Hospital, Bloorview Research Institute, Toronto, Canada; 4 Institute of Health Policy, Management & Evaluation, University of Toronto, Toronto, ON Canada; 5 Rehabilitation Sciences Institute, University of Toronto, Toronto, ON Canada

**Keywords:** Integrated knowledge translation, Ikt, Patient engagement, Multidisciplinary collaboration, Multiple sclerosis, Research co-creation, Research partnership, Knowledge user

## Abstract

**Background:**

Integrated knowledge translation (iKT) represents an approach to optimizing health interventions research through active collaboration between researchers and knowledge users throughout the research process. To date, few studies have explored the process of engaging in iKT, particularly in the context of multiple sclerosis (MS) research. Building on a larger iKT-informed study exploring mindfulness-based interventions for people living with MS, this study explores the perspectives of iKT panellists and extended collaborators on the use of iKT in health research.

**Methods:**

This qualitative descriptive study utilized one-on-one semi-structured interviews conducted using Zoom or Microsoft Teams. Interviews were 20–30 min in duration. An interview guide informed by the Ontario Brain Institute’s framework for patient engagement across the stages of research was used. Interviews were transcribed verbatim, coded, and analyzed using inductive thematic analysis.

**Results:**

A total of eight iKT partners were interviewed, five were members of the iKT panel and three were extended collaborators. Five themes on the use of iKT in health interventions research on MS were identified: (1) defining iKT, (2) motivation and meaningful participation in iKT, (3) the importance of networking in iKT, (4) balancing multiple perspectives, and (5) barriers and facilitators to engaging in iKT. Within these themes, interviewees highlighted the need for further definition and operationalization of concepts. Discussion on the representativeness of iKT partners and recruitment of ‘hard to reach’ knowledge users was also salient.

**Conclusion:**

The findings from this study provide useful considerations for other teams using an iKT approach. Future research directions include finding/maximizing meaningful ways for knowledge users to participate, exploring ways in which knowledge users could lead/co-lead (rather than consult on) research activities, and examining the potential role of an iKT facilitator.

**Supplementary Information:**

The online version contains supplementary material available at 10.1186/s40900-025-00763-7.

## Abstract (plain language summary)

Integrated knowledge translation (iKT) in health interventions research is an approach that involves actively collaborating with a variety of people such as patients, health care providers, and policy decision-makers. This means working with these groups at each stage of the research process (e.g., designing a study, collecting data, analyzing and interpreting data, and sharing the results of the study). By conducting research this way, the interventions produced from these studies are thought to be more relevant, effective, and long-lasting for everyone.

However, the process of doing this type of research has not been thoroughly explored, especially in the context of multiple sclerosis (MS) research. Therefore, we interviewed a variety of people with experience conducting or contributing to this kind of research. In these interviews, many highlighted the need for further clarification of terminology. Discussions on who tends to participate in iKT research and whether their perspectives represent the ‘typical’ experience also occurred during interviews.

The findings from this study provide useful considerations for other teams that are interested in using an iKT approach. Future research directions include finding and maximizing meaningful ways for people to participate, exploring ways in which non-researchers can lead/co-lead (rather than consult on) research activities, and examining the potential role of an iKT facilitator.

**Supplementary Information:**

The online version contains supplementary material available at 10.1186/s40900-025-00763-7.

## Introduction

Integrated knowledge translation (iKT) represents an approach to optimizing the implementation of interventions such as MBIs through active collaboration between researchers and a variety of knowledge users including patients, clinicians, and decision-makers [1, 2]. This entails dynamic and iterative engagements with these key informants across various stages of the research process [3]. By engaging with a diverse set of knowledge users, interventions can be tailored to better meet the needs of multiple groups, leading to better outcomes in practice [1–4]. Various reviews on the use of iKT in research have demonstrated the importance of this approach in generating relevant, effective, and sustainable interventions [1, 2]. 

Given the proposed benefits of iKT, this approach is growing in uptake among health researchers. This has led to increasing accounts and reflections on knowledge user engagement, such as discussions on barriers and facilitators to using iKT [3, 5]. However, few studies have focused on the process of conducting iKT-informed research, and fewer have focused on engagement involving a variety of disability knowledge users. Strategies to promote sustained relationships that are integral to future iKT activities also represents a gap in the literature. Knowing that each stage of the research process (i.e., conception, recruitment, data collection, analysis, and dissemination) poses unique challenges, the engagement of a variety of knowledge users can add further complexity [1]. As the importance of using iKT becomes increasingly recognized and efforts to improve patient engagement in research increase over time, this study begins to address gaps in knowledge around the experiences of knowledge users that have engaged in iKT.

This study builds upon a larger study evaluating the use and implementation of mindfulness-based interventions (MBIs) for PwMS informed by an iKT approach. Given the main study’s use of an iKT approach, this created a unique opportunity to qualitatively examine the process of using iKT in health interventions research. This was achieved through semi-structured interviews exploring the perspectives of a multi-knowledge user iKT panel, as well as extended collaborators on their experiences contributing to iKT across the research process. The iKT panel consisted of PwMS, clinicians, and researchers bringing both lived experience and professional academic experience. The panel provided input on activities at each stage of the research study, as well as participated in the execution of activities.

## Methods

### Study design and paradigm

This qualitative descriptive study explored the use of iKT throughout each stage of the health research process. This was deemed appropriate given the focus on understanding knowledge users’ experiences with the iKT process. This study was grounded in a pragmatic approach, focusing on the practicality of applying an iKT approach to health interventions research [6]. 

The Ontario Brain Institute’s framework for patient engagement across the stages of research was used to develop topics discussed during interviews [7]. This framework outlines the potential steps that researchers can implement during each research stage such as determining the relevance of research to patients and the public, advising on the vocabulary used in study materials, and providing feedback [7]. The stages of research outlined in this framework, as well as the steps suggested in each stage, were used to form items in the semi-structured interview guide.

Approval to conduct this study was received from the University Health Network research ethics board (UHN REB #23-5748). This study was reported as per the COREQ (COnsolidated criteria for REporting Qualitative research) and GRIPP2-LF checklists.

### Recruitment of participants

Members of the iKT panel and extended collaborators who provided input on the larger study were contacted by email to participate (*n* = 11). Two knowledge users did not respond, and one did not participate due to their involvement in the planning of this study and the larger study, leading to a perceived conflict of interest. In total, a volunteer sample of eight interviewees were recruited. These were comprised of PwMS, clinicians, and researchers. All recruited interviewees provided written informed consent (physical and electronic signatures were accepted) and were aware of the objectives of this study.

### Data collection

Interviewees completed a one-on-one semi-structured interview using a virtual platform (Zoom or Microsoft Teams). Interviews took approximately 20–30 min and were conducted from August to October 2024. Basic sociodemographic information (gender and number of years in practice, if applicable) was collected. An interview guide developed by GF, DL, SM, and RS was then used to facilitate the discussion. The interview guide began with broad items on definitions of iKT and motivations for using an iKT approach. This was followed by items on knowledge user implementation of iKT in each stage of the research process (planning, recruitment and retention, study execution, data analysis, dissemination). The interview guide concluded with items on developing sustained relationships with knowledge users and future directions. Exploring the full range of research activities allowed for maximization of “information power” by collecting a large volume of data from the sample [8]. The full interview guide is included in Appendix 1.

Interviews were conducted by GF, who had contact with some iKT partners as a member of the research team for the larger study. GF is a male research analyst with an MPH in epidemiology and three years of experience conducting qualitative research. All interviews were recorded and transcribed verbatim. Transcripts were not returned to interviewees for feedback.

### Data analysis

The accuracy of transcripts was verified by the interviewer (GF) and inductive thematic analysis was conducted as described by Braun and Clarke [9]. Briefly, this included (1) familiarization, (2) coding, (3) initial theme generation, (4) reviewing and developing themes, (5) refining, defining, and naming themes, and (6) producing the report [9]. Transcripts were coded by GF and a subset of these transcripts were reviewed by SM and RS (principal and co-principal investigators). Codes were then grouped to form overarching themes. Through biweekly meetings, GF, SM, and RS explored various coding trees until a consensus was reached and theme labels were agreed on. The final results from the analysis are described within this manuscript, which was co-authored by a patient partner living with MS who also has a background in research.

## Results

**Participants**.

A total of eight people were interviewed, which included researchers, clinicians, clinician/ researchers, health service leaders, and people living with MS who also had a background in research. Five were part of the multi-knowledge user iKT panel and three were extended collaborators. Six were women and two were men. Among researchers, clinicians, and clinician/researchers, the median number of years in practice was 11 years (ranging from 4 to 31 years).

### Theme 1: Defining iKT

As iKT partners discussed their understandings and working definitions of iKT, the following three sub-themes were identified: (1) dynamic and multidisciplinary collaboration, (2) importance of identifying relevant knowledge users, and (3) ambiguous use of iKT terminology and jargon. Representative quotes are presented in Table 1.

#### Dynamic and multidisciplinary collaboration

The importance of multidisciplinary collaboration (i.e., working with a range of knowledge users from academia to applied practice, including patient populations and care partners) to intentionally incorporate a wide variety of perspectives was emphasized as a core component of iKT. Interviewees also discussed iKT as a “two-way dialogue” between researchers and the people who use and benefit from research. Some also defined iKT as a strategy to promote knowledge sharing amongst different groups of people across the research process.

#### Importance of identifying relevant knowledge users

iKT partners identified a variety of potential knowledge users to engage with throughout the health research process. Examples of knowledge users included PwMS, care partners, clinicians, researchers, and policy-makers. In the context of interventions research, emphasis was also placed on engaging with knowledge users involved in the financial aspects of implementing a health intervention. Understanding the key players was framed as essential to ensuring that the products of an iKT project would be both relevant and useful to those involved.

#### Ambiguous use of iKT terminology and jargon

Although most interviewees identified similar objectives and components of iKT, some were not familiar with terms such as “knowledge users” and “integrated”. This led to some confusion in providing a definition of iKT. In some cases, distinctions between knowledge translation and integrated knowledge translation seemed ambiguous to interviewees, and conflation of the two terms was identified.

**Table 1:** Sub-themes and quotes branching from the main theme of defining iKT

Theme	Sub-theme	Quote
Defining iKT	Dynamic and multidisciplinary collaboration	“It is a collaborative effort to understand some phenomena and so you’re not just having researchers on the iKT panel… you’re trying to get some insight with people that are knowledge users” (Participant 1, PwMS)
		“My understanding is it’s a dialogue, a two way dialogue between people who produce the research and people who use the research.” (Participant 3, Clinician/Researcher)
	Importance of identifying relevant knowledge users	“It’s important to talk to care partners… people forget that they’re very involved often with all stages of their [PwMS’] care… definitely people in the healthcare space, like clinicians, specifically their primary care physician, psychiatrists, OTs, PTs, anyone who’s involved” (Participant 5, Researcher)
		“Definitely having them [PwMS] involved is really important so that we’re not creating something for them without actually getting their voice.” (Participant 4, Researcher)
		“You do need to have people that are paying for those things, like the insurances, the payers, the policy makers, you need to have the scientists to provide the evidence in a proper manner that everybody will understand.” (Participant 3, Clinician/Researcher)
	Ambiguous use of iKT terminology and jargon	“To be honest, iKT is not a concept that I particularly interact with on a day-to-day basis. I don’t have much of a working definition for it… So again, you’re asking some pretty abstract questions” (Participant 2, Clinician)

### Theme 2: Motivation and meaningful participation in iKT

iKT partners described a variety of reasons for participating in the iKT panel, as well as ways in which participation could be made more meaningful. This is explored in three sub-themes: (1) personal and professional connections to the topic, (2) opportunities to exchange knowledge, (3) building motivation to contribute to iKT through connection and belonging. Representative quotes are presented in Table 2.

#### Personal and professional connections to the topic

Several iKT partners cited personal and professional connections to the topic of interest (i.e., mindfulness and MS), thereby influencing their decision to participate in the iKT panel. Some iKT partners had previously referred their own patients to mindfulness programs, while others had connections to the co-investigators involved in the main study.

#### Opportunities to exchange knowledge

Opportunities to exchange knowledge were frequently cited as reasons for participating in the iKT panel. This included gaining new perspectives and providing one’s own perspectives. For instance, one participant noted that fewer opportunities exist for clinicians to contribute to research activities, and thus the iKT panel offered a platform for them to share their expertise with a diverse set of individuals. When collaborating with people living with MS or patients in general, providing them with the opportunity to participate in scientific writing and dissemination activities was also discussed by a clinician/researcher as a way to help establish their ‘authority’ as experts.

#### Building motivation to contribute to iKT through connection and belonging

PwMS were identified as a knowledge user group that can be harder to recruit for iKT panel membership that subsequently entails contributing to various stages of a research study. In this regard, some interviewees perceived that motivation to join an iKT panel could be built among this group through connection and belonging. For instance, the iKT panels could provide opportunities for PwMS to use their lived experience to shape interventions that would help other PwMS. iKT partners also expressed that being a part of iKT panels fostered a sense of belonging among PwMS by working with other knowledge users towards a shared goal.

**Table 2:** Sub-themes and quotes branching from the main theme of motivation and meaningful participation in iKT

Theme	Sub-theme	Quote
Motivation and meaningful participation in iKT	Personal and professional connections to the topic	“For this particular panel, I was involved with the research project itself and the formation of the project… but also my own interests in MS, having people in my life who have had MS, and also being in the mental health and well-being space…” (Participant 5, Researcher)
	Opportunities to exchange knowledge	“Clinicians like myself often feel, you know, researchers are out there doing all this cool stuff, and we’re down here doing all this cool stuff, but there’s, there’s very little opportunity to interact” (Participant 2, Clinician)
		“And being part of the scientific writing makes them [patient partners] an author and an authority. So now when they speak in public, they have the authority because they were an author of the paper.” (Participant 3, Clinician/Researcher)
	Building motivation to contribute to iKT through connection and belonging	“I think people often seek to make meaning out of their diagnosis, so really emphasizing for them how their contributions have helped others in the MS community, and how their time and efforts are being translated. I think those can be very meaningful motivators for patients.” (Participant 6, Clinician)
		“They already feel that our society has excluded them because of their disability. When you show interest in them, and not just interest because I want to collect your data, but you know, “tell me about your life, share your experience with us, we can give you your data back at the end of the study, we’ll let you know about webinars, meetings” when, when they feel they part of this bigger process, of part of the big intellectual endeavour, they stay with you. Showing genuine interest in people’s lived experiences.” (Participant 7, Clinician/Researcher)

### Theme 3: The importance of networking in iKT

Across the stages of the research process, the importance of networking was framed as both a means to conduct iKT activities as well as an outcome of engaging in iKT. The following sub-themes were identified: (1) professional networks, (2) patient networks, (3) relationship building. Representative quotes are presented in Table 3.

#### Professional networks

Researchers in the process of building an iKT panel described relying on pre-existing professional connections with knowledge users, and using those connections to find other potential members. Moreover, researchers described leveraging these connections to conduct research activities such as participant recruitment for research studies. In this regard, professional networks were framed as a useful resource to enable iKT activities.

#### Patient networks

Many interviewees cited the importance of harnessing patient networks across the research process. As some PwMS are active members of patient support groups, identifying those with strong community membership can be instrumental in accessing these groups. In the early stages of a study, PwMS could enable snowball sampling or facilitate the recruitment process by sharing information with their networks. During the dissemination stage of a study, researchers and clinicians described how PwMS can help share research findings through peer-to-peer communication. This could help to enhance the credibility of research activities among PwMS while empowering PwMS to lead the narrative around how new research findings are framed post-publication.

#### Relationship building

Networking was also discussed as an outcome of iKT. If relationships with knowledge users are thoughtfully developed and sustained, this can lead to further collaboration on future research projects. This can also help to foster a sense of comradery and belonging within the iKT panel which was believed to improve the outputs and overall quality of the research project.

**Table 3:** Sub-themes and quotes branching from the main theme of the importance of networking in iKT

Theme	Sub-theme	Quote
The importance of networking in iKT	Professional networks	“Oftentimes we engage people that are in our sort of sphere of net, like known researchers or networks, depends on the on the research topic as well. Oftentimes, we might ask a colleague to refer someone to us that we may not know. I think it’s often driven by the study itself or the project.” (Participant 7, Researcher)
	Patient networks	“I do know that there’s a couple of patients who are sort of like almost leaders … they like run their own like coping groups, or they volunteer supporting other patients with MS… if there’s a way to reach those people it may be helpful to try to disseminate through them” (Participant 6, Clinician)
		“And how can you make the data available to the broader public, what’s the best way of putting it out there?… For example, the person with MS can be a spokesman… this can be very, very helpful, because people listen to those who’ve got lived experience.” (Participant 8, Clinician/Researcher)
	Relationship building	“Because then that could also lead to further support down the road. So I think that’s another part of that relationship building, is that if you can penetrate their network…” (Participant 7, Researcher)

### Theme 4: Balancing multiple perspectives

The logistics of engaging with a variety of knowledge users was discussed during several interviews. Through these discussions, two sub-themes were identified: (1) the potential role of facilitators and (2) knowledge user-specific needs. Representative quotes are presented in Table 4.

#### The potential role of facilitators

Many researchers explored the idea of having a dedicated facilitator who would lead iKT panel meetings. As coordinating and engaging with a variety of knowledge users can be time consuming and complex, this could alleviate some of the pressure placed on researchers. At the same time, the importance of having a neutral facilitator was also discussed. Ensuring that the facilitator is not affiliated with a specific knowledge user group (i.e., researchers) could help to reduce implicit power imbalances between researchers ‘leading’ the study and other knowledge users in the panel.

#### Knowledge user-specific needs

iKT partners frequently cited differences in the needs of different knowledge user groups. Clinicians discussed the need for practical outputs of iKT-informed projects such as protocols or clinical practice guidelines outlining how clinicians can apply findings to their work. Others perceived that policy-makers often needed faster timelines and larger outputs, which could be challenging given the pace of traditional research projects.

**Table 4:** Sub-themes and quotes branching from the main theme of balancing multiple perspectives

Theme	Sub-theme	Quote
Balancing multiple perspectives	The potential role of facilitators	“We are putting in more effort now in patient engagement because it’s become so important. But I think it’s an extremely time consuming thing to do. And I almost feel like there’s needs to be someone… who actually is the glue that holds the whole [iKT] team together… like it’s not up to the researcher anymore, because that’s so much work.” (Participant 7, Researcher)
		“Having somebody on your team who’s always willing to be by their email or their phone or whatever it might be to answer their [knowledge users’] call is really important. And maybe that would even be having the person, like, an arm’s length from the research team, so they don’t feel like they’re calling a researcher and have to justify themselves for why there’s a disagreement” (Participant 4, Researcher)
	Knowledge user-specific needs	“For people like myself, oftentimes we would love to get our protocols right. So if there’s a study that doesn’t just say, like, you know, we did this magical thing, and look it worked, but here’s exactly what we did. It worked.” (Participant 2, Clinician)
		“In research, we have a research question, and then we go design a study to answer that question. For policymakers, they want to know what impact your research will have, how soon, and what resources are needed, and how quickly can you get it…” (Participant 7, Researcher)

### Theme 5: Barriers and facilitators to engaging in iKT

Several barriers and facilitators to engaging in iKT were explored during interviews. These led to the sub-themes of: (1) ‘hard to reach’ knowledge users, (2) finding the right mix, (3) coordinating multiple schedules, and (4) attitudes and perceptions. Representative quotes are presented in Table 5.

#### ‘Hard to reach’ knowledge users

Some knowledge user groups were identified as being harder to reach and recruit for iKT activities. PwMS were identified as ‘hard to reach’ due to potential self-selection/selection biases (e.g., considering who would volunteer to participate, or have the time and resources to participate). Furthermore, policy makers were also identified as being hard to reach due to higher levels of turnover in the policy field.

#### Finding the right mix

Finding the right mix of knowledge user groups on a panel was cited as a challenge in conducting iKT. While having a large number of panellists could help to ensure that a variety of perspectives are incorporated, it could also create practical challenges in integrating a large volume of perspectives and finding a compromise that suits a variety of needs.

#### Coordinating multiple schedules

The importance of providing flexible options to participate in iKT was discussed by multiple interviewees. Having a larger number of panel members often created challenges in finding a unanimous meeting time. Therefore, hosting virtual meetings and allowing knowledge users to provide feedback asynchronously was key to allowing all panel members to engage.

#### Attitudes and perceptions iKT panellist skillsets

Across interviews, researchers held conflicting perspectives about which stages of the research process warrant more engagement with an iKT panel and how the panel should be engaged. For instance, during the analysis stage, some researchers suggested that iKT panellists should review research outputs after they have been compiled as they may not be able to contribute beyond reviewing. Others felt that panellists should be given the opportunity to look at raw data and engage in writing alongside research personnel.

**Table 5:** Sub-themes and quotes branching from the main theme of barriers to engaging in iKT

Theme	Sub-theme	Quote
Barriers and facilitators to participating in iKT	‘Hard to reach’ knowledge users	“I think sometimes patient representatives can self-select and have a different sort of… not represent the whole sample of potential users.” (Participant 6, Clinician)
		“A very difficult group that we often like to involve, but difficult to recruit, are policy makers, policy or decision makers… there’s a high turnover” (Participant 7, Researcher)
	Finding the right mix	“It’s always a balance, right? Because you don’t want to have a panel that’s too big and too many people putting in their input, but maybe trying to have people with MS from different disability perspectives or levels or phenotypes, because they might have totally different perspectives on what we’re doing.” (Participant 4, Researcher)
	Coordinating multiple schedules	“Providing flexible options to provide their expertise so either through email communications so that way they kind of do it on their own time… doing a phone call versus Zoom call… being able to tailor it to their needs based on each individual” (Participant 5, Researcher)
	Attitudes and perceptions of iKT panellist skillsets	“…it would be more in the interpretation stage, like after some of this has been done, to kind of go back and confirm” (Participant 1, PwMS)
		“Okay, here are the raw tables, and then help me try to interpret, help me to get the main message. Help me to see what is the story here that you want me to tell?” (Participant 3, Clinician/Researcher)

## Discussion

Through interviews with eight knowledge users, five themes on the use of iKT in health interventions research on MS were identified. These themes included: (1) defining iKT, (2) motivation and meaningful participation in iKT, (3) the importance of networking in iKT, (4) balancing multiple perspectives, and (5) barriers and facilitators to participating in iKT.

### Defining iKT

Among those familiar with iKT, there appeared to be some consensus around the definition and objectives of iKT (e.g., involving dynamic and multidisciplinary collaboration across the research process). This overlaps with previous scoping reviews on iKT which have found that involvement of diverse knowledge users throughout the research process is central to conducting iKT-informed research and ultimately producing higher quality outputs [1, 2]. However, among knowledge users with less exposure to iKT, there was some confusion around terminology and meaning. For instance, differentiating between knowledge translation and iKT, and definitions of knowledge users.

These discrepancies can make it challenging for knowledge users that are less familiar with the terminology to engage, calling the inclusivity of iKT-informed research into question. At the same time, this ambiguity could also undermine the cohesion of an iKT panel if members have differing expectations. In a previous review, lack of knowledge on iKT and consensus on the objectives of conducting iKT-informed research were found to be barriers to using an iKT approach [1]. In this regard, further efforts to use plain language in discussions with knowledge users and to define frequently used jargon at the onset of a study is warranted. Similarly, to foster meaningful engagement, research teams are encouraged to devote time to explaining what is meant by iKT and the different ways that knowledge users can be involved in the research process.

### Motivation and meaningful participation in iKT

Several panel members cited personal and professional connections to the research topic as reasons for participating in the iKT panel. iKT partners also discussed how iKT panels could be used to build a sense of connection and belonging, particularly among PwMS, which could further strengthen their motivation to engage. In a study exploring the effects of participation in a youth advisory group on health research, interviewees commonly cited a desire to make ‘meaningful’ contributions on matters concerning them, and to have an impact on research related to their age group [10]. Knowing the importance of building connection, further exploration of how researchers can intentionally create these opportunities for iKT panellists to meaningfully engage is needed [11]. Moreover, reframing iKT to not only focus on the benefits for researchers, but to intentionally create benefits for patients and other knowledge users is warranted to ensure that these partnerships are not only for the direct benefit of researchers [12]. 

### The importance of networking in iKT

Findings within this theme highlighted the role of networking as both a process and an outcome of iKT. Professional networks were identified as a means to recruit members for an iKT panel, as well as to accomplish certain aspects of the research process such as participant recruitment. Patient networks were similarly identified as a means to recruit participants, as well as to disseminate the results of a study to PwMS. Finally, interviewees highlighted that by building strong relationships with knowledge users, further collaboration in future research projects can be made possible. This overlaps with a previous study on iKT involving a school-based project, that highlighted the importance of building supportive relationships with knowledge users to foster a culture of trust in the research process [13]. In this regard, fostering an environment where knowledge users such as PwMS can form connections with other knowledge users is one way to sustain engagement over time, while building knowledge, capacity, and ultimately creating efficiencies in how knowledge users can collaborate on research projects.

### Balancing multiple perspectives

In order to integrate feedback from multiple knowledge users, ensure equal distribution of power in the iKT process, and reduce the pressure placed on researchers, the potential role of a designated iKT facilitator was discussed. For instance, Phoenix et al. reflect on who ‘owns the table’, influences who gets to ‘sit at the table’, and what gets discussed [14]. Often, it is researchers who are in control of iKT activities throughout a research study. This raises the question of who should facilitate iKT activities and which background they should bring to the role. As other studies have suggested that external facilitation can promote mutual respect and equal partnership [15], and in some cases having multiple facilitators may be needed [16], further investigation into the role of facilitation in iKT is warranted.

In interviews with knowledge users, discrepancies between the knowledge needs of clinicians, policy-makers, and researchers were identified. For instance, in interviews with clinicians, outputs such as a protocol were emphasized. Alternatively, interviewees described policy-makers as needing faster product cycles than a traditional research project would allow for. This overlaps with other studies on iKT, which described a disconnect between the timelines in a research study and the outputs that clinicians may be looking for [17]. This emphasizes the importance of working with knowledge users to establish a common set of objectives at the beginning of a research project, and continuing to revisit these objectives and expectations over the course of the project.

### Barriers and facilitators to engaging in iKT

Engaging with ‘hard to reach’ knowledge users such as PwMS and policy-makers was identified as a barrier to iKT. Moreover, many iKT partners noted that the types of knowledge users who agree to participate in iKT may not be representative of the broader group. As noted by Phoenix et al., knowledge users who are invited to be research partners are often of higher socioeconomic status and share more similarities than differences with co-investigators [14]. In this regard, the perspectives of certain knowledge users can be missed. Similarly, interviewees described challenges in finding the right number and mix of knowledge users to engage with. While engaging with a large volume of knowledge users could improve the utility of the final product, it could also create challenges in integrating all perspectives. Further exploration into ways to engage with ‘hard to reach’ knowledge users and the optimal balance of perspectives is warranted.

Additional barriers such as the logistics of coordinating multiple schedules were identified. While asynchronous alternatives such as allowing for knowledge users to provide feedback over email were described, these alternatives may limit opportunities for knowledge users to connect with each other and participate in discussions. Moreover, the typical iKT panel format may not be optimal to allow knowledge users to take on roles in leadership over a project, thereby limiting their sense of connection to the work. This barrier has been identified in several projects involving iKT [1, 18]. In one study, some knowledge users were reluctant to commit to long-term participation or in activities that could interfere with leisure/personal time [18]. At the same time, reducing the roles of knowledge users in an iKT-informed study has the potential to diminish the important input provided by knowledge users and create reluctance to contribute long-term. Compensation represents another way in which this barrier could be addressed.

Attitudes and perspectives towards when knowledge users should be engaged, the level of engagement required in each stage of a research study, and the nature of their involvement was discussed. While some interviewees suggested that knowledge users should provide consultation at specific points of the research process, others felt that their participation could be more fluid and active. Knowing that knowledge users can be engaged across all stages of research [2], this highlights the implicit assumptions that can be made around the role of knowledge users. Further work exploring the engagement of an iKT panel in specific activities (e.g., study design, data collection, data analysis) outside of consultation, notably in co-leadership roles, is needed. It also raises the importance of discussing with knowledge users at the outset of a research project the specific activities they would like to be involved in and their level of involvement (e.g., advisor versus co-leader). Notably, some studies have suggested that researchers may be reluctant to maximize engagements with knowledge users out of fear of losing control over the research process [18]. Conversely, lack of trust among community members toward researchers has also been identified as a barrier in other studies [19]. In this regard, the attitudes and perceptions of both researchers and other knowledge users can hinder iKT activities.

### Strengths and limitations

This study has several strengths. It is one of the first studies to explore the use of iKT in the context of health interventions research for PwMS, thereby beginning to fill a gap in the current literature. It is also one of the first studies to explore iKT across the research process, drawing from both members of an iKT panel and extended collaborators with experience in this work. Moreover, this study explored the experiences of knowledge users with exposure to iKT, which furthers the conversation on practical approaches to using iKT in MS research. Finally, interviewees in this study belonged to a variety of knowledge user groups including PwMS, clinicians, researchers, and clinician/researchers.

We also acknowledge some limitations, including a smaller sample size. Moreover, despite interviewing members from a variety of knowledge user groups, only one participant was a PwMS. We were unable to recruit caregivers and policy-makers, which are two important knowledge user groups whose perspectives should also be considered. This limits how the findings from this study can be used to enhance engagement with these knowledge user groups. As interviewees in this study were mainly clinicians and researchers affiliated with a major metropolitan university/hospital, their perspectives may not be transferable to the broader health research environment.

## Conclusions

Although there is some consensus regarding the definition and purpose of iKT, interviewees in this study also highlighted the need for further definition and operationalization of concepts. Interviewees also highlighted the representativeness of iKT panellists and recruitment of ‘hard to reach’ knowledge users as key barriers to conducting iKT. Future research directions include finding/maximizing meaningful ways for a variety of knowledge users to participate, exploring ways in which knowledge users could co-lead the research process rather than consult on it, and examining the potential role of an iKT facilitator.

## Electronic supplementary material

Below is the link to the electronic supplementary material.

Supplementary Material 1

Supplementary Material 2

Supplementary Material 3

## Data Availability

The qualitative data supporting the findings of this study (i.e., full transcripts) are stored within the University Health Network, but restrictions apply to the availability of these data due to the smaller sample size and risk of identification. Upon reasonable request, interested parties may contact the authors for this data. With the permission of interview participants and the University Health Network REB, data may be released on a case-by-case basis.
