# Supplementary material for: Knowledge user perspectives on integrated knowledge translation (iKT) in health interventions research for people with multiple sclerosis: a qualitative descriptive study
Source: Res Involv Engagem. 2025 Jul 28;11:88. doi: 10.1186/s40900-025-00763-7 (PMC12306047; doi:10.1186/s40900-025-00763-7)
Supplement: Supplementary file 2 — Supplementary Material 2 [file 40900_2025_763_MOESM2_ESM.pdf]

GRIPP2 Long Form (GRIPP2-LF) Reporting Checklist

Section and topic	Item	Reported on page No
**Section 1: Abstract of paper**		
1a: Aim	Report the aim of the study	1
1b: Methods	Describe the methods used by which patients and the public were involved	1
1c: Results	Report the impacts and outcomes of PPI in the study	1
1d: Conclusions	Summarise the main conclusions of the study	1
1e: Keywords	Include PPI, "patient and public involvement," or alternative terms as keywords	2
**Section 2: Background to paper**		
2a: Definition	Report the definition of PPI used in the study and how it links to comparable studies	3
2b: Theoretical underpinnings	Report the theoretical rationale and any theoretical influences relating to PPI in the study	3
2c: Concepts and theory development	Report any conceptual or theoretical models, or influences, used in the study	3
**Section 3: Aims of paper**		
3: Aim	Report the aim of the study	3
**Section 4: Methods of paper**		
4a: Design	Provide a clear description of methods by which patients and the public were involved	3-4
4b: People involved	Provide a description of patients, carers, and the public involved with the PPI activity in the study	4
4c: Stages of involvement	Report on how PPI is used at different stages of the study	5
4d: Level or nature of involvement	Report the level or nature of PPI used at various stages of the study	5
**Section 5: Capture or measurement of PPI impact**		
5a: Qualitative evidence of impact	If applicable, report the methods used to qualitatively explore the impact of PPI in the study	4

5b: Quantitative evidence of impact	If applicable, report the methods used to quantitatively measure or assess the impact of PPI	N/A
5c: Robustness of measure	If applicable, report the rigour of the method used to capture or measure the impact of PPI	N/A
**Section 6: Economic assessment**		
6: Economic assessment	If applicable, report the method used for an economic assessment of PPI	N/A
**Section 7: Study results**		
7a: Outcomes of PPI	Report the results of PPI in the study, including both positive and negative outcomes	7-10
7b: Impacts of PPI	Report the positive and negative impacts that PPI has had on the research, the individuals involved (including patients and researchers), and wider impacts	7-13
7c: Context of PPI	Report the influence of any contextual factors that enabled or hindered the process or impact of PPI	12-13
7d: Process of PPI	Report the influence of any process factors, that enabled or hindered the impact of PPI	10-13
7ei: Theory development	Report any conceptual or theoretical development in PPI that have emerged	N/A
7eii: Theory development	Report evaluation of theoretical models, if any	N/A
7f: Measurement	If applicable, report all aspects of instrument development and testing (eg, validity, reliability, feasibility, acceptability, responsiveness, interpretability, appropriateness, precision)	N/A
7g: Economic assessment	Report any information on the costs or benefit of PPI	N/A
**Section 8: Discussion and conclusions**		
8a: Outcomes	Comment on how PPI influenced the study overall. Describe positive and negative effects	13-16
8b: Impacts	Comment on the different impacts of PPI identified in this study and how they contribute to new knowledge	13-17

8c: Definition	Comment on the definition of PPI used (reported in the Background section) and whether or not you would suggest any changes	14
8d: Theoretical underpinnings	Comment on any way your study adds to the theoretical development of PPI	13-17
8e: Context	Comment on how context factors influenced PPI in the study	16
8f: Process	Comment on how process factors influenced PPI in the study	16
8g: Measurement and capture of PPI impact	If applicable, comment on how well PPI impact was evaluated or measured in the study	N/A
8h: Economic assessment	If applicable, discuss any aspects of the economic cost or benefit of PPI, particularly any suggestions for future economic modelling.	N/A
8i: Reflections/critical perspective	Comment critically on the study, reflecting on the things that went well and those that did not, so that others can learn from this study	13-16
