# Supplementary material for: Knowledge user perspectives on integrated knowledge translation (iKT) in health interventions research for people with multiple sclerosis: a qualitative descriptive study
Source: Res Involv Engagem. 2025 Jul 28;11:88. doi: 10.1186/s40900-025-00763-7 (PMC12306047; doi:10.1186/s40900-025-00763-7)
Supplement: Supplementary file 3 — Supplementary Material 3 [file 40900_2025_763_MOESM3_ESM.docx]

**APPENDIX 1**

**Semi-structured Interview Guide**

Thank you for taking the time to participate in this interview. As a knowledge user, we are interested in learning about your perspectives on integrated knowledge translation (iKT) and the ways in which iKT can be applied while studying mindfulness-based interventions for people with multiple sclerosis. Although this interview will be recorded and analyzed by our research team, personally identifying information such as names will be removed from the transcript. Do you have any questions before we begin?

1. iKT has been broadly defined as an active and dynamic collaboration between researchers and knowledge users in the conduct of health research. How would you define iKT? Does this fit your understanding of iKT?
   1. How might other people define iKT?
   2. How would you define a knowledge user?
2. What motivated you to participate in our iKT panel?
   1. What has made you continue participating?
   2. Have there been any barriers to participating?
3. How would you determine whether research on mindfulness interventions for people living with MS is relevant to potential knowledge users?
   1. Why might people living with MS be interested in a mindfulness intervention? What might be some reasons why they wouldn’t be interested?
   2. How could we engage with knowledge users in the planning stage for this specific study? Which knowledge users might be most helpful?
4. What role could knowledge users have in the recruitment and retention of clients with MS, caregivers, or other relevant knowledge users (e.g., clinicians, instructors, etc.) in a mindfulness study?
   1. Which knowledge users might be most helpful in this stage? How could we enable participation in this process?
   2. What might be some of the challenges in recruiting people with MS for a mindfulness intervention?
5. When conducting a study at the data collection stage, what are some ways that knowledge users could be involved?
   1. How should data from people living with MS be collected (e.g., interviewing, closed-ended questionnaires, online, etc.)? Why?
   2. After completing a mindfulness intervention, which outcomes should be measured and why? How should we prioritize outcomes of interest?
6. When the data collection process has been completed, how should knowledge users be engaged in the data analysis process?
   1. Should knowledge users be involved in analyzing data, interpreting the results of an analysis, or both?
   2. Are there opportunities for disagreements between researchers and knowledge users during the analysis stage? How could these disagreements be resolved?
7. When the results from a study have been finalized (e.g., in a manuscript, report, etc.), where should the results be shared in order to reach knowledge users?
   1. How do you think knowledge users typically find information on mindfulness or other research studies? How might this vary by knowledge user type?
   2. How could the results of a study on mindfulness be disseminated to knowledge users that do not have a research background?
   3. What improvements, if any, could be made in how researchers typically share their results with knowledge users?
8. How could we build lasting or sustained relationships with MS knowledge users?
   1. Which knowledge users may be harder to reach or retain? How could we ensure that patient partners aren’t burdened by their participation in the research process?
   2. How can we support patient partners or other knowledge users that have less time, energy, or expertise to participate in research?
   3. What would make participation in an iKT panel more meaningful for participants? Or from your perspective, what do you think constitutes meaningful participation?

Thank you for your time today. Is there anything else you would like to add that we haven’t covered in the interview today? Are there any important points you would like to re-emphasize?
